# Factors influencing the benefits of pulmonary rehabilitation in older adults with chronic obstructive pulmonary disease: a prospective study

**DOI:** 10.3389/fpubh.2025.1644607

**Published:** 2025-09-17

**Authors:** Li Feng, Qing-Qing Yang, Mengyao Liang

**Affiliations:** Department of Nursing, The Sixth People's Hospital of Nantong, Nantong, Jiangsu, China

**Keywords:** COPD, pulmonary rehabilitation, older adults, benefits, influencing factors

## Abstract

**Objective:**

To investigate the benefits of pulmonary rehabilitation (PR) in older adults with stable chronic obstructive pulmonary disease (COPD) and analyze its influencing factors.

**Methods:**

From January 2023 to April 2024, convenience sampling was adopted to collect 254 stable patients with COPD who met the inclusion and exclusion criteria in a Classiii hospital in Nantong City as the research objects. Among them, 196 patients completed the PR course and were finally included in the study. According to the PR benefit criteria, they were divided into the benefit group and the non-benefit group, and the incidence of benefit and influencing factors were analyzed. The generalized estimating equations (GEE) were used to plot the trend profiles of physical capacity and quality of life before rehabilitation and at 3, 6, and 9 weeks after rehabilitation.

**Results:**

A total of 196 patients completed the PR course, 107 patients achieved PR benefit, benefit at a rate of 54.59%. Logistic regression analysis showed that, Chronic pain (OR = 0.43, 95%Cl:0.22 ~ 0.83, *p* = 0.011), baseline 6MWD (OR = 0.98, 95%Cl:0.96 ~ 0.99, *p* < 0.001), sarcopenia (OR = 0.50, 95%Cl:0.27 ~ 0.95, *p* = 0.035), better economic situation (OR = 1.96, 95%Cl:1.03 ~ 3.71, *p* = 0.039), and good family care index (OR = 2.11, 95%Cl:1.08 ~ 4.11, *p* = 0.029) were the influencing factors of pulmonary rehabilitation benefits in older adults with stable COPD.

**Conclusion:**

The PR benefit rate of patients with clinically stable COPD is low, which is mainly affected by the baseline 6MWD, chronic pain, sarcopenia, economic situation, and family care index. Clinical medical staff should consider the influencing factors when they perform PR for patients.

## Introduction

Chronic obstructive pulmonary disease (COPD) is characterized by persistent airflow limitation, decreased exercise capacity, and quality of life and is often progressive (1). According to research, the number of Chinese patients with COPD will increase from 88.3 million to 103.3 million (2) and predicts that 3.9 million people will die from COPD between 2020 and 2039 (2), thus imposing a significant economic and social burden on China.

The 2024 Global Initiative for COPD proposes a strategy that includes both drug and non-drug therapies to manage COPD symptoms and prevent disease progression, to improve the quality of life for a long time. Among them, pulmonary rehabilitation (PR) (3) is considered an important part of non-drug treatment and for the management of COPD. However, it has been reported (4, 5) that although the PR program is well organized, some patients still fail to benefit from exercise capacity, quality of life, and other aspects after completing the relevant rehabilitation courses. Therefore, it is essential to understand the benefits of PR in patients with COPD to improve the outcomes of PR programs and properly utilize the existing clinical rehabilitation resources (6). Currently, research on the benefits of PR in patients with COPD is primarily concentrated in Europe and the United States, It is important to perform research in other countries for a better understanding of the disease. The influencing factors include age, dyspnea grade, arterial oxygen partial pressure, comorbidities, and body mass index (4, 7, 8), and the results show a certain degree of heterogeneity. Due to regional differences in population characteristics and medical environments, previous research results may not apply to our country.

The Chinese COPD population exhibits distinct geographic and culturally-specific phenotypic characteristics. Firstly, national epidemiological survey data show that rural patients in China are widely exposed to biomass fuels, with individuals exposed to biomass fuels demonstrating a significantly higher risk of developing COPD (OR = 2.58) compared to non-exposed individuals (9, 10). The fibrotic processes in small airways induced by such exposure fundamentally differ from smoking-induced damage alone (11). Secondly, the familial support system shaped by Confucian culture (e.g., multi-generational households) profoundly influences rehabilitation behaviors, manifesting as higher rates of accompanied medical visits and treatment supervision frequency (12, 13). This kinship-based collective health management model markedly differs from Western individualistic rehabilitation paradigms. Additionally, and most importantly, inherent biological differences in muscle metabolism among Asian populations—evidenced by ethnically specific diagnostic criteria established through evidence-based medicine (14) (e.g., grip strength cutoffs: Asian males < 28.0 kg vs. European/American < 32.5 kg)—combined with grain-dominated dietary patterns leading to insufficient essential amino acid intake, collectively form a unique risk profile for sarcopenia development in Chinese COPD patients (15, 16).

This exploratory cohort study aimed to find independent predictors of PR response based on the medical environment and cultural characteristics of older adults COPD patients in China. Based on the Chinese characteristics of COPD patients and other studies on the benefits of pulmonary rehabilitation, we hypothesized that: (1) patients with lower baseline exercise capacity (6MWD) were more likely to benefit from PR; (2) Sarcopenia or chronic pain may reduce the efficacy of PR; (3) Family support and economic status may be protective factors for treatment adherence.

## Objects and methods

### Participants

In this prospective study, a convenience sampling strategy was used to select stable patients with COPD in a Class III general hospital in Nantong City, Jiangsu Province as the survey objects from January 15, 2023 to April 20, 2024. Inclusion criteria: Patients specifically diagnosed with COPD according to “China’s guidelines of diagnosis and treatment of chronic obstructive pulmonary disease (2020 revision)” (17), patients who participated in the respiratory center’s outpatient PR program, had no symptoms of acute exacerbation in the first 4 weeks, were in a stable condition, and were 60 to 89 years old, receiving regular drug therapy, permanent residents of Nantong (living in Nantong for more than half a year), with clear consciousness and, no language communication obstacles were included in the study. Patients with other respiratory diseases, mental illness, or serious heart, liver, or kidney disease; patients with motor dysfunction, Patients who did not complete the corresponding PR courses (less than 70%); and patients with incomplete information, such as those whose PR program could not be reviewed were excluded from the study. Patients were divided into a benefit group (achieving both: ≥30 m 6MWD and ≥4-point SGRQ) and non-benefit group. In this study, binary Logistic regression analysis was used to screen the influencing factors of pulmonary rehabilitation. The sample size calculation was based on the following parameters: with 6MWD as the primary outcome measure, the minimum clinically important difference (MCID) for 6MWD in patients with COPD was 30 m. G*Power 3.1 software was used for *a priori* analysis, and the test power (1-*β*) was set as 0.8, the significance level *α* = 0.05, and the effect size Cohen’s d = 0.5 (moderate effect). At least 128 samples were needed for prediction. Sixteen independent variables were included in this study. According to the rule, the sample size should be 5 to 10 times the number of independent variables. An initial sample size of 160 cases was considered for this study. Considering a 10–20% rate of invalid questionnaires (18), at least 178 cases were required for the study. The study was approved by the Ethics Committee of the Sixth People’s Hospital of Nantong (approval number: NTLYLL2023015), and all patients provided written informed consent.

### Completion criteria and benefit criteria of PR program for patients

#### PR plan and completion criteria

Follow-up after PR for hospitalized patients: All patients were comprehensively assessed by clinicians before starting the PR program. The clinicians, who did not participate in the late stages of rehabilitation, conducted the assessment at the respiratory center clinic. Upon the first complete assessment, exclusive medical records were prepared for each patient. The PR program lasted for 9 weeks with 2 to 3 sessions per week accounting for a total of 18 to 24 treatment sessions. Specific intervention plans were prepared according to the severity of the disease, which included exercise therapy, self-management skills, etc. sports training: includes aerobic exercise, strength training, and balance training, aiming to improve the patient’s muscle strength, endurance, and balance ability, show in Supplementary File 1. Self-management skills, including breathing training, medication management, nutritional guidance and psychological support, are designed to help patients better manage their disease, reduce symptoms and improve quality of life. Patients were contacted by telephone the night before the start of the course to confirm their admission to the hospital and participation in the rehabilitation program. If the patients participated normally, this was recorded. The term “admission to hospital” refers to the patient’s arrival at the respiratory rehabilitation clinic and initiation of a pulmonary rehabilitation program. Patients who confirmed their participation in the program but failed to attend on time were considered as not having participated in a regular manner. If they did not participate normally, they were provided with relevant course videos via WeChat for online learning, in addition to exercise training. At the next time of admission, the patients were asked oral questions to assess their level of mastery. If they had not mastered the material, they were re-taught on the spot. Patients returned to the respiratory center every 3 weeks to measure their 6-min Walk Distance (6MWD). For patients who were not hospitalized on time but still required on-site guidance for PR, COPD “Internet + nursing service” was provided through mobile phone. Relevant research on this approach has been published in appropriate journals in China (19–21). Participants who completed >70% (22) of prescribed sessions were classified as ‘complete’ to ensure intervention fidelity and mitigate efficacy assessment bias from inadequate adherence.

#### PR benefit criteria

Currently, there is no gold standard for judging the benefits of PR. Based on a high-quality systematic review (23), this study adopted exercise capacity and quality of life improvement as benefit outcome indicators. The 6MWD was used to evaluate the patients’ exercise ability, and the St. George’s Respiratory Questionnaire (SGRQ) was used to evaluate the patient’s quality of life. Referencing previous studies (4, 8), the minimal clinically important difference criteria of increase in 6MWD and decrease in SGRQ were used the benefit (24, 25). In this study is defined as an improvement in 6MWD of ≥ 30 m and a reduction in SGRQ score of ≤ − 4 units before and after the PR program.

### Survey tools

#### General information questionnaire

PubMed, Cochrane Library, CNKI, Wanfang, VIP, CBM, and other databases were searched for relevant literature on the influencing factors of PR in COPD. Variables were screened based on clinical and expert opinions. A general information questionnaire was prepared, including details about age, drinking habits, smoking status, body mass index (BMI), marital status, education level, living situation, economic situation, and whether there was the presence of osteoporosis. Based on the per capita disposable income in Nantong for 2022 (26), an income of less than 4,091 yuan per month was defined as poor economic status, while an income of more than 4,091 yuan per month was defined as better economic status. The presence of osteoporosis was determined by asking patients directly or consulting relevant case data.

#### 6-min walk distance test procedure

The 6MWD was performed under the supervision of medical personnel. The test was performed to measure the distance a person could walk within 6 min while experiencing shortness of breath (27). The specific procedure was to walk in a 30-meter-long corridor with flat, straight, and hard surfaces, wearing comfortable clothes and shoes while the patients were instructed to remain calm before the test. During the test, patients walked at their maximum tolerated speed for 6 min. If the patient experiences intolerance, the speed is allowed to be slowed down or paused, and the test could be resumed after recovery. Medical personnel were present at all times to monitor the patients. Patients were motivated with standard encouraging phrases, such as “You are doing well” and “Keep up the good work”.

### Sarcopenia

According to the criteria recommended by the 2019 Asian Working Group on Sarcopenia (AWGS) (28), factors such as muscle strength, limb skeletal muscle mass index (RASM), and low physical performance should be considered in the evaluation of sarcopenia. (1) When measuring grip strength, participants were asked to hold the dynamometer as hard as they could to evaluate the handedness and non-dominant hand grip strength (kg) using dynamometer evaluation. According to 2019 AWGS standards (28), for men a grip strength < 28 kg, and for women a grip strength < 18 kg is considered as low muscle strength. (2) The formula used to calculate RASM is limb skeletal muscle mass (kg)/height (m)^2^, and limb skeletal muscle mass (ASM) refers to the following formula:

ASM = 0.193 * weight (kg) + 0.107 * height (cm) − 4.157 * gender − 0.037 * age (years) − 2.631. Weight was measured by Omron TMHN-286 scale, height was measured by SecaTM213 altimeter, and gender was set to 1 if the patient was male and 0 if the patient was female. Several studies have demonstrated that ASM calculated by this formula is in good accordance with the dual-energy X-ray absorbent (DXA) method (28). If a female had RASM ≤ 5.7 and RASM ≤ 7 in males along with low muscle strength, it was considered as confirmed sarcopenia. (3) Gait speed was measured by timing the patients while walking 50 meters. If the gait speed was less than 1.0 m/s, the patient was considered to have low physical function. The formula may overestimate the muscle mass of patients with edema (such as right heart failure), and is only recommended as an alternative by AWGS (when DXA/BIA is unavailable).

### St. George’s Respiratory Questionnaire

St. George’s Respiratory Questionnaire (SGRQ) was used to evaluate the quality of life of patients from three main aspects of health including symptoms, mobility, and disease impact. The scores range from 0 to 100. A higher score indicates worse health status of patients. A reduction of 4 or more units in the SGRQ score between groups is considered clinically significant. The Cronbach’s *α* coefficient for the SGRQ in patients with COPD is 0.98 (29).

### Modified dyspnea index

The modified Medical Research Council (mMRC) dyspnea index is a widely used tool in clinical practice to measure the severity of dyspnea in patients (30). The scale is a 5-point scale. A score of 0 indicates difficulty in breathing only during strenuous activity and increasing in turn, and a score of 4 indicates severe difficulty in breathing while leaving the house, or breathlessness when dressing or undressing.

### BODE index

The BODE index is a multidimensional tool used to assess the prognosis and severity of COPD. To evaluate disease impact, it integrates 4 key components including BMI (B), degree of airway obstruction (O), degree of dyspnea (D), and exercise capacity (E) (31). The sum of the four points is the BODE index, with lower scores indicating better conditions. A score of 0 to 2 indicates mild disease condition, 3 to 4 indicates moderate, 5 to 6 indicates severe, and a score of 7 to 10 extremely severe Table 1.

**Table 1:** BODE index grading.

Project	0 points	0 points	2 points	3 points
FEV_1_%pre (%)	≥65	50 ~ 64	36 ~ 49	≤35
6MWT (m)	≥350	250 ~ 349	150 ~ 249	≤149
mMRC	0 ~ 1	2	3	4
BMI (kg/m^2^)	>21	≤21		

### Family care index

The Family Care Index (Family APGAR Index, APGAR) (32), is a member of the Family subjective evaluation tool for Family satisfaction. It is scored based on five items. Each item is scored on a scale of 0 to 2, where 2 indicates “normal,” 1 indicates “sometimes” and 0 indicates “rarely.” The total score ranges between 0 to 10 points. A score of 7 to 10 is considered good family functioning, and a score of 0 to 6 is considered a severely dysfunctional family. The scale of Cronbach’s alpha coefficient is 0.80 ~ 0.88, and is widely used in the family in China, and has demonstrated good validity in assessing family satisfaction.

### Data collection and quality control method

Data was collected by five trained personnel during the PR program. The BMI, 6MWD test, SGRQ, mMRC, and BODE index scores were recorded before the start of the PR program. The 6MWD and SGRQ were re-evaluated and recorded in the medical record system after the PR course by the evaluators who did not participate in the PR program. The researchers conducted a thorough check and verification of data collected by two researchers to ensure its completeness, authenticity, and accuracy.

### Statistical methods

SPSS 26.0 statistical software was used for data analysis. Patient age, 6MWD results, SGRQ scores, and mMRC scores were found to conform to a normal distribution. The mean and standard deviation describe; gender, drinking, smoking, and body mass index (BMI). Other classification data descriptions included frequencies and composition ratios. *χ*^2^ test, t-test, and Mann-Whitney U test were used for univariate analysis. Logistic regression analysis was used to identify factors associated with the benefits of PR in patients. The significance level was set at alpha = 0.05. Sensitivity analyses included three levels: (1) mandatory inclusion of demographic variables (Model 1); (2) increasing indicators of disease severity (Model 2); (3) Multiple imputation analysis (Model 3). All analytic variables including age, gender, 6MWD, SGRQ, mMRC scores, and smoking status. Twenty imputed datasets were generated using chained equations. The Fully Conditional Specification algorithm was implemented with predictive mean matching for continuous variables and logistic regression for categorical variables. Convergence was confirmed after 50 iterations per chain when autocorrelation function values fell below 0.1. All procedures were executed in SPSS 26.0 MVA module with random seed fixed at 202305.

The generalized estimating equation (GEE)was used to analyze the 6MWD and SGRQ scores of patients in the benefit group and the non-benefit group before rehabilitation and at the end of 3, 6, and 9 weeks of rehabilitation, and the trend profile was drawn. The GEE method can effectively incorporate information on missing data to estimate model parameters, eliminating the need for imputation or deletion of missing data.

## Results

### General information and PR benefit status of the research subjects

**Figure 1:**
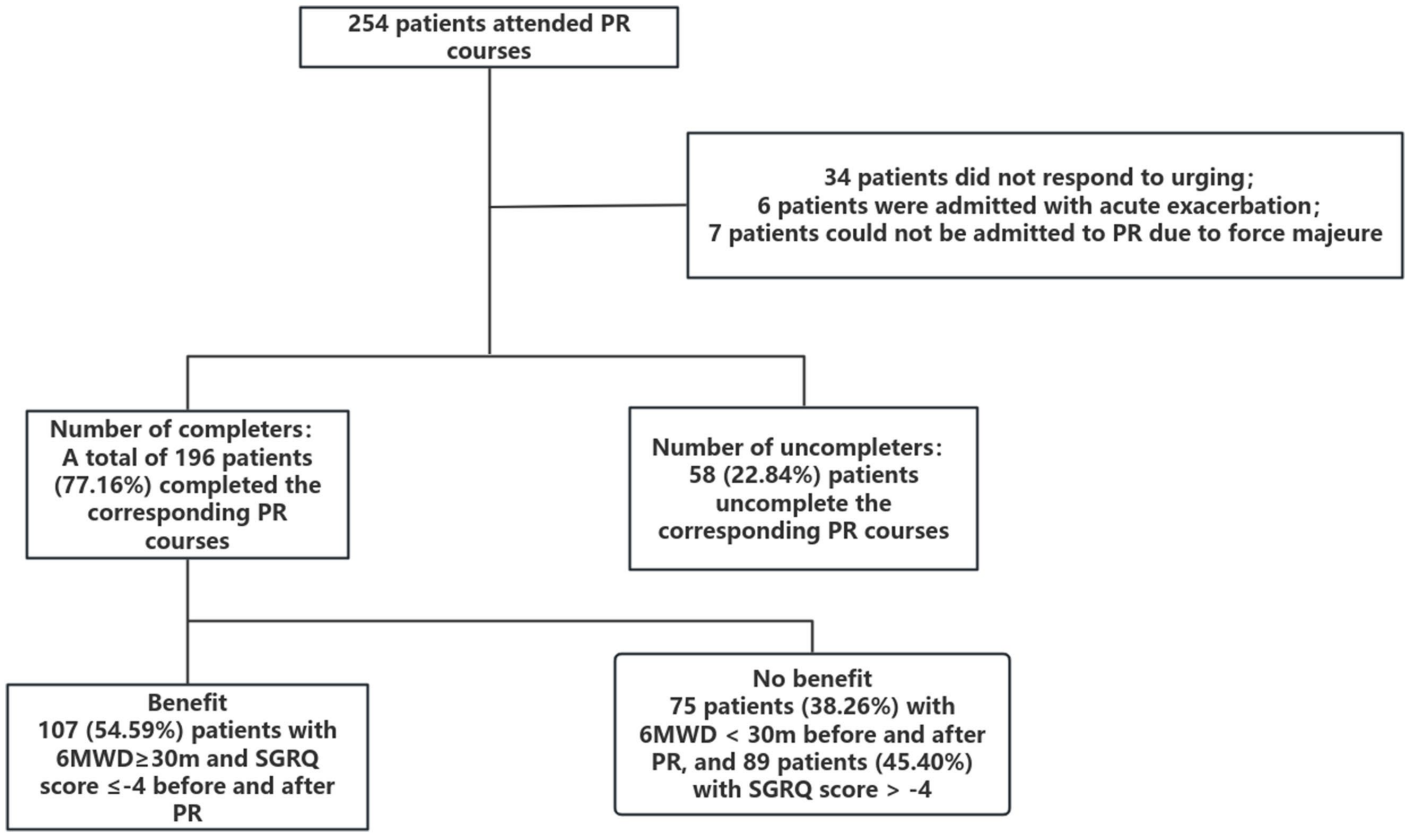
PR flow chart.

Among 254 initially enrolled patients with COPD, 196 (77.2%) completed the PR program. 58 patients (22.8%) discontinued participation. Detailed temporal patterns of missing data (baseline, 3-week, 6-week, 9-week) and Little’s MCAR test results (*χ*^2^ = 7.32, *p* = 0.29) are provided in Supplementary Table S1. 6 patients were readmitted due to acute exacerbation, 34 patients did not respond to the urging, 7 patients could not be admitted to the hospital due to force majeure factors, and 4 patients did not respond. 11 cases did not have corresponding 6MWD or SGRQ evaluations, as shown in Figure 1. Among the 196 completers, 107 cases (54.59%) met the benefit criteria (an increase of ≥ 30 meters in 6MWD and a reduction of ≥ 4 points in SGRQ), 121 patients only met the 6MWD improvement standard, and 107 patients only met the SGRQ improvement standard.

A prospective cohort study was conducted with 196 patients aged 60 to 87 years (mean age 73.18 ± 6.59 years). Univariate analysis identified several baseline factors including 6MWD, chronic pain, sarcopenia, economic situation, and APGAR to be statistically significant (*p* < 0.05), Table 2.

**Table 2:** Univariate analysis of pulmonary rehabilitation benefits in patients with COPD [cases (percentage, %)].

Variables	Total (*n* = 196)	Non benefit (*n* = 89)	Benefit (*n* = 107)	Statistic	*p*
Baseline 6MWD (m)	325.82 ± 26.68	333.180 ± 20.24	319.71 ± 29.77	*t* = 3.751	< 0.001
Baseline SGRQ (points)	41.94 ± 4.96	41.57 ± 4.98	42.25 ± 4.95	*t* = −0.953	0.342
mMRC (points)	2.06 ± 0.74	2.07 ± 0.77	2.04 ± 0.73	*t* = 0.297	0.767
Age (years)				*χ*^2^ = 4.487	0.106
Mean ± SD	73.18 ± 6.59	73.82 ± 7.93	72.13 ± 8.20	*t* = 1.461	0.146
60 ~ 69	92 (46.94)	37 (41.57)	55 (51.40)	*χ*^2^ = 4.487	0.106
70 ~ 79	79 (40.30)	36 (40.45)	43 (40.19)		
80~	25 (12.76)	16 (17.98)	9 (8.41)		
Marriage, *n* (%)				*χ*^2^ = 0.095	0.758
Married	169 (86.22)	76 (85.39)	93 (86.92)		
Divorced/widowed	27 (13.78)	13 (14.61)	14 (13.08)		
Education level, *n* (%)				*χ*^2^ = 1.590	0.452
Primary school and below	52 (26.53)	20 (22.47)	32 (29.91)		
Middle School and High School	115 (58.67)	54 (60.67)	61 (57.01)		
College above	29 (14.80)	15 (16.85)	14 (13.08)		
Smoking status, *n* (%)				*χ*^2^ = 2.839	0.242
Never	36 (18.37)	13 (14.61)	23 (21.50)		
Once	134 (68.37)	61 (68.54)	73 (68.22)		
At present	26 (13.26)	15 (16.85)	11 (10.28)		
Alcohol consumption, *n* (%)				*χ*^2^ = 5.187	0.075
Never	42 (21.43)	16 (17.98)	26 (24.30)		
1 times a month or less	95 (48.47)	39 (43.82)	56 (52.34)		
More than once a month	59 (30.10)	34 (38.20)	25 (23.36)		
BMI (kg/m^2^)					
Mean ± SD	21.4 ± 3.2	21.2 ± 3.4	21.6 ± 3.0	*t* = 0.893	0.372
<18.5	57 (29.08)	26 (29.21)	31 (28.97)	*χ*^2^ = 0.004	0.998
18.5~	115 (58.67)	52 (58.43)	63 (58.88)		
24~	24 (12.25)	11 (12.36)	13 (12.15)		
BODE, *n* (%)				*χ*^2^ = 1.488	0.475
Mild	86 (43.87)	35 (39.33)	51 (47.66)		
Moderate	91 (46.42)	44 (49.44)	47 (43.93)		
Severe	19 (9.69)	10 (11.23)	9 (8.41)		
Chronic pain, *n* (%)				*χ*^2^ = 5.993	0.015
No	128 (65.31)	50 (56.18)	78 (72.90)		
Yes	68 (34.69)	39 (43.82)	29 (27.10)		
Sarcopenia, *n* (%)				*χ*^2^ = 7.045	0.008
No	119 (60.71)	45 (50.56)	74 (69.16)		
Yes	77 (39.29)	44 (49.44)	33 (30.84)		
Status of residence, *n* (%)				*χ*^2^ = 0.314	0.575
Cities	99 (50.51)	43 (48.315)	56 (52.336)		
Township	97 (49.49)	46 (51.685)	51 (47.664)		
Economic situation (RMB)				*χ*^2^ = 6.031	0.015
<4,091	109 (55.61)	58 (65.16)	51 (47.66)		
≥4,091	87 (44.39)	31 (34.84)	56 (52.34)		
APGAR, *n* (%)				*χ*^2^ = 4.863	0.028
Bad	76 (38.78)	42 (47.20)	34 (31.77)		
Good	120 (61.22)	47 (52.80)	73 (68.23)		
Osteoporosis, *n* (%)				*χ*^2^ = 0.240	0.624
No	113 (57.65)	53 (59.55)	60 (56.07)		
Yes	83 (42.35)	36 (40.45)	47 (43.93)		

*t*: t-test, *χ*^2^: Chi-square test.

Age and BMI data presented both as mean ± SD (t-test) and categorical distributions (*χ*^2^ test).

### Multiple factor analysis of benefits from PR in patients with COPD

The study investigated whether the benefits of PR could be predicted by various baseline factors. The significant variables from the univariate analysis were included in a binary logistic regression analysis. The specific assignment of each variable is shown in Table 3. Logistic regression analysis identified five significant predictors of PR response (Table 4). Chronic Pain (OR = 0.43, 95% CI 0.22 ~ 0.83; *p* = 0.011), indicating that patients with chronic pain demonstrated a 57% reduction in the probability of benefit (1-OR) compared to those without pain; Sarcopenia (OR = 0.50, 95% CI 0.27 ~ 0.95; *p* = 0.035), suggesting that sarcopenic patients exhibited half the probability of benefit attainment; Economic Advantage (OR = 1.96, 95% CI 1.03 ~ 3.71; *p* = 0.039), showing that economically stable participants had nearly double the likelihood of benefit; Family Support (APGAR: OR = 2.11, 95% CI 1.08 ~ 4.11; *p* = 0.029), indicating that high family support doubled the probability of benefit; Baseline 6MWD (OR = 0.98, 95% CI 0.96 ~ 0.99; *p* < 0.001), with each 10-meter increment in baseline walking distance conferring an 18% reduction in benefit odds (1–0.98^10^).

**Table 3:** PR benefit variable assignment table for patients with COPD.

Variables	Assigned values
PR benefit	0 = Nonbenefit; 1 = Benefit
Chronic pain	0 = No; 1 = Yes
sarcopenia	0 = No; 1 = Yes
Economic situation (RMB)	0 = <4,091; 1 = ≥4,091
Baseline exercise capacity (6MWD)	Continuous variable (m)
APGAR	0 = bad; 1 = good

**Table 4:** Logistic regression analysis of influencing factors of pulmonary rehabilitation benefits in patients with COPD.

Variables	Single factor regression					Multivariate regression				
	*β*	S. E	*Z*	*p*	OR (95%CI)	*β*	S. E	*Z*	*p*	OR (95%CI)
Chronic pain	−0.741	0.305	−2.430	0.015	0.48 (0.26 ~ 0.87)	−0.846	0.334	−2.535	0.011	0.43 (0.22 ~ 0.83)
Sarcopenia	−0.785	0.298	−2.635	0.008	0.46 (0.25 ~ 0.82)	−0.685	0.324	−2.114	0.035	0.50 (0.27 ~ 0.95)
Economic situation (good)	0.720	0.295	2.441	0.015	2.05 (1.15 ~ 3.66)	0.672	0.326	2.061	0.039	1.96 (1.03 ~ 3.71)
APGAR good	0.652	0.297	2.194	0.028	1.92 (1.07 ~ 3.43)	0.746	0.341	2.189	0.029	2.11 (1.08 ~ 4.11)
Baseline 6MWD (m)	−0.020	0.006	−3.415	<0.001	0.98 (0.97 ~ 0.99)	−0.024	0.007	−3.527	<0.001	0.98 (0.96 ~ 0.99)

### Comparison of regression models with sensitivity adjustments

Effect sizes remained stable (< 5% change) for chronic pain (OR = 0.45 vs. 0.43) and sarcopenia (OR = 0.52 vs. 0.50) after adjustment for age, sex, and BODE index, as shown in Table 5. In addition, to validate the potential impact of baseline 6MWD on the efficacy evaluation, baseline 6MWD was mandatorily included as a covariate in the GEE analysis. After adjusting for baseline 6MWD, the time trend in the benefit group (*β* = 4.1 vs. original 4.3) and the independent effect of baseline walk distance (*β* = −0.12/m, *p* < 0.001) remained significant. For every 1-m increase in baseline, the subsequent improvement in 6MWD decreased by 0.12 m, suggesting a persistent influence of baseline functional status on the rehabilitation trajectory, as shown in Table 6.

**Table 5:** Comparison of regression models with sensitivity adjustments.

Variable	Model 1 OR (95%CI)	Model 2 OR (95%CI)	Model 3 OR (95%CI)
Chronic pain	0.45 (0.23 ~ 0.88)	0.44 (0.22 ~ 0.85)	0.42 (0.21 ~ 0.84)
Sarcopenia	0.52 (0.28 ~ 0.97)	0.51 (0.27 ~ 0.96)	0.49 (0.26 ~ 0.93)
Economic status	1.92 (1.01 ~ 3.65)	1.89 (1.02 ~ 3.62)	1.98 (1.04 ~ 3.77)
APGAR	2.09 (1.07 ~ 4.08)	2.05 (1.05 ~ 4.02)	2.14 (1.09 ~ 4.20)
6MWD (per m)	0.98 (0.96 ~ 0.99)	0.98 (0.96 ~ 0.99)	0.98 (0.96 ~ 0.99)

Model 1: Original model + Age (continuous) + Sex (male/female); Model 2: Model 1 + BODE index; Model 3: Multiple imputation model (*n* = 254).

**Table 6:** GEE analysis results after adjusting for baseline 6MWD.

Parameter	β (SE)	Adjusted *p* value	Original model β (SE)	Unadjusted *p*-value
6MWD model				
Benefit group	13.8 (3.3)	< 0.001	15.2 (3.1)	< 0.001
Time (every 3 weeks)	7.9 (1.6)	< 0.001	8.7 (1.5)	< 0.001
Group × time interaction	4.1 (1.0)	< 0.001	4.3 (0.9)	< 0.001
Baseline 6MWD (per meter)	−0.12 (0.03)	< 0.001		

### Change trajectories of exercise capacity and quality of life in patients with COPD between the benefit group and non-benefit group during PR

This study utilized GEE to analyze repeated measures data. The model specifications were as follows: (1) an exchangeable correlation structure was selected as the optimal working correlation matrix based on the Quasi-Likelihood Independence Criterion (QIC = 328.7 vs. 335.2 for unstructured and 341.5 for autoregressive); (2) an identity link function with Gaussian distribution was applied for the continuous dependent variables (6MWD and SGRQ).

**Figure 2:**
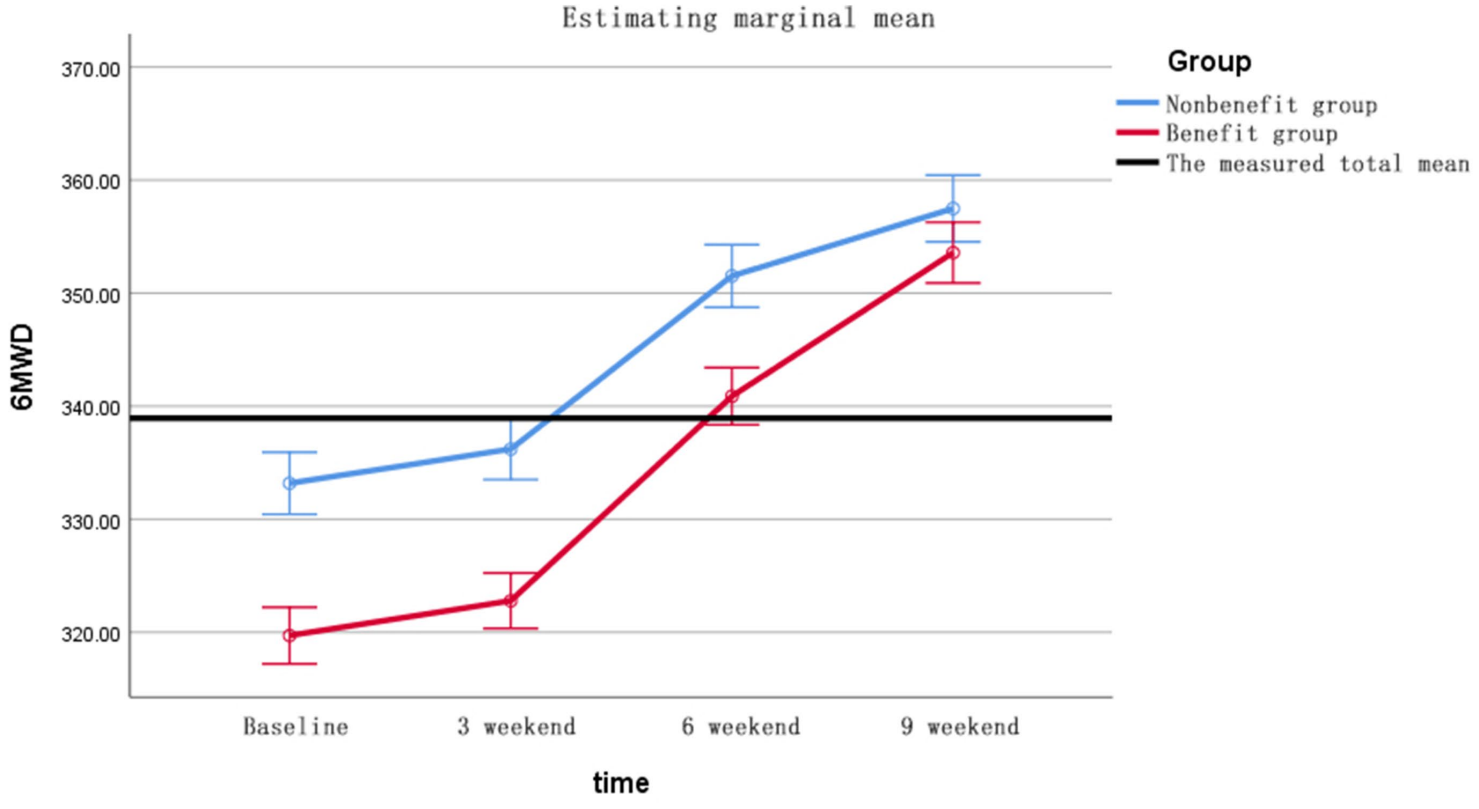
The 6MWD trend profile of the two groups of patients.

**Figure 3:**
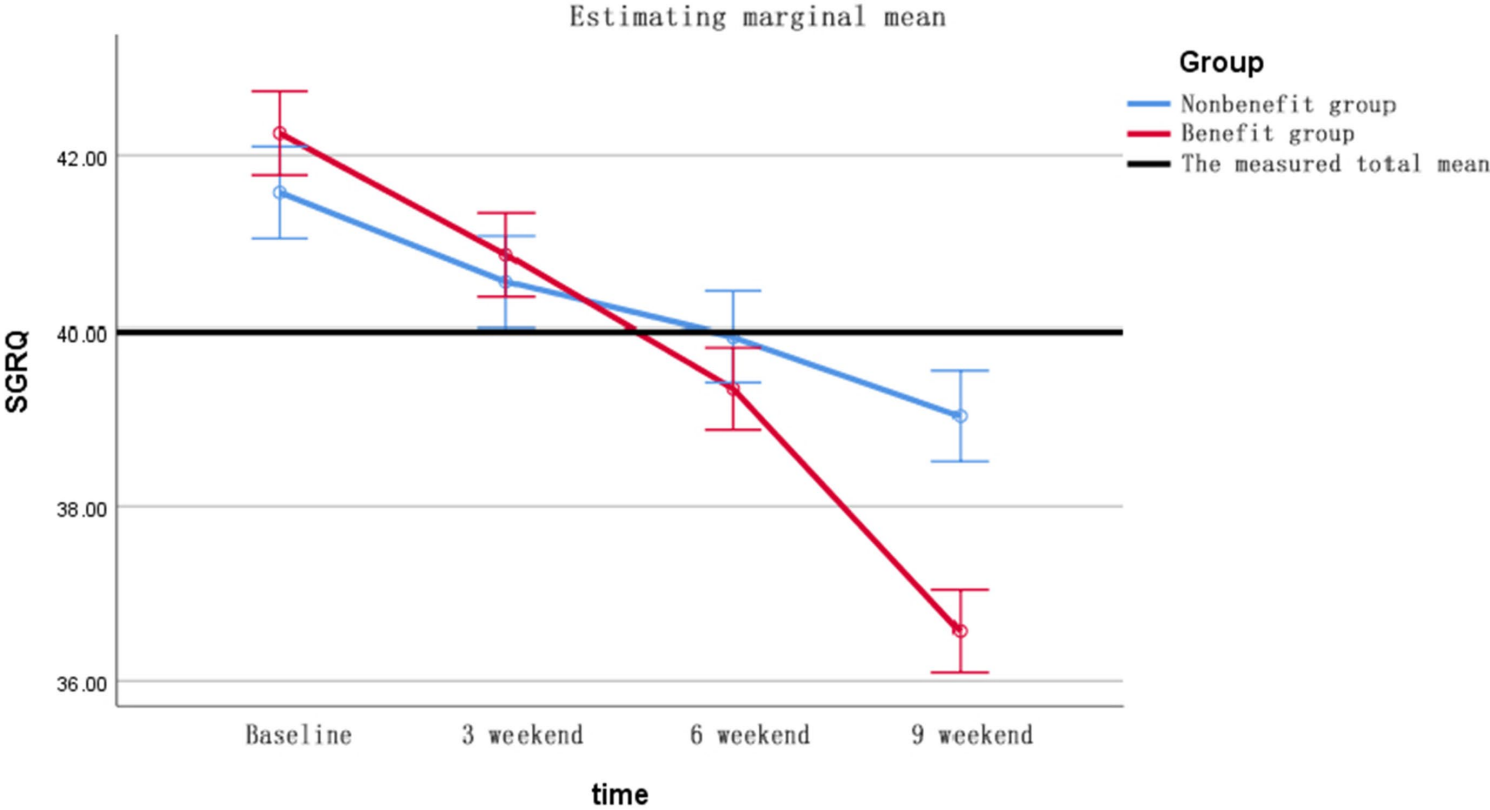
SGRQ trend profiles of the two groups of patients.

**Figure 4:**
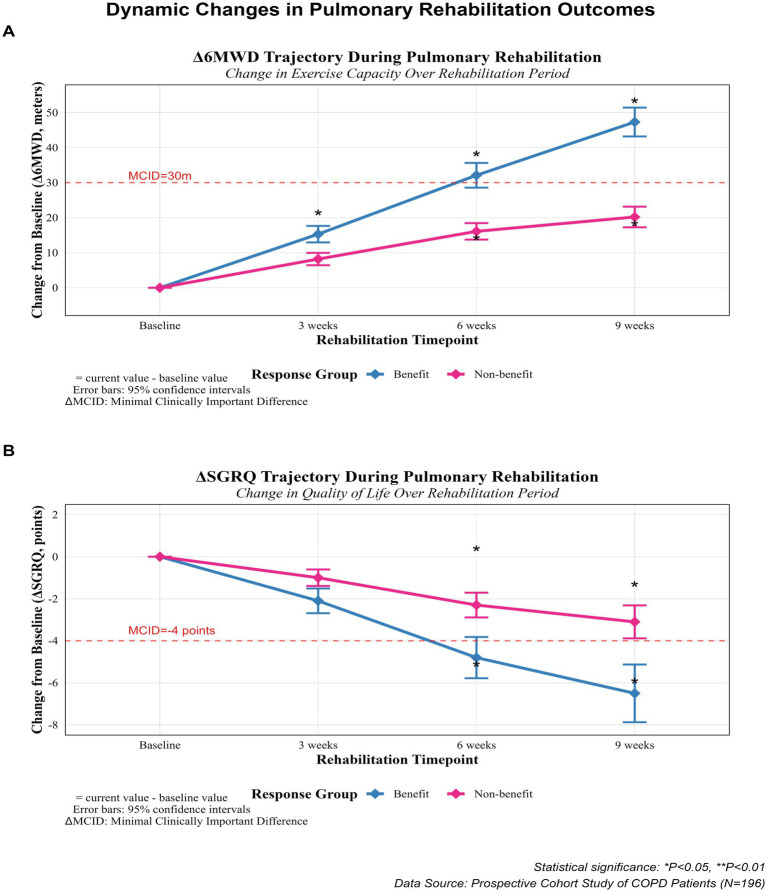
The changes in *Δ*6MWD and ΔSGRQ during pulmonary rehabilitation between the benefit group and the non-benefit group. Δ= current value - baseline value; *p < 0.05.

6MWD changes (Figure 2) GEE showed significant time effect (Wald *χ*^2^ = 67.3, *p* < 0.001), between-group difference (*χ*^2^ = 14.8, *p* < 0.001) and time-by-group interaction (*χ*^2^ = 8.9, *p* = 0.003). Changes in SGRQ (Figure 3) shows the group main effect (*χ*^2^ = 11.2, *p* = 0.001), time effect (*χ*^2^ = 53.7, *p* < 0.001) and interaction effect (*χ*^2^ = 5.9, *p* = 0.015), and the specific improvements are shown in Table 7. The changes in Δ6MWD and ΔSGRQ during pulmonary rehabilitation in the benefit group and the non-benefit group are shown in Figure 4.

**Table 7:** Improvement at different time points in each group.

Variables	Group	Baseline	3 weeksΔ	6 weeksΔ	9 weeksΔ
6MWD(m)	Benefit	319.7 ± 29.8	+ 15.3^**^	+ 32.1^**^	+ 47.3^**^
	Nonbenefit	333.2 ± 20.2	+ 8.2^*^	+ 16.1^**^	+ 20.2^**^
SGRQ(points)	Benefit	42.2 ± 4.9	− 2.1^**^	− 4.8^**^	− 6.5^**^
	Nonbenefit	41.6 ± 4.9	− 1.0^*^	− 2.3^**^	− 3.1^**^

Δ = current value-baseline,^*^p < 0.05, ^**^p < 0.01.

## Discussion

### Low benefit rate of PR in patients with COPD

As a comprehensive intervention measure, PR includes exercise training, health education, and strategies to promote behavior change, etc. Evidence indicates that (30) these measures will have a positive impact on reducing the rate of readmission and mortality of patients with COPD. However, some studies have shown that at the end of the PR, nearly one-third or one-half of the patients do not show significant improvements in terms of sports ability and/or quality of life (5, 7). This research shows that at the end of a nine-week PR course, the benefit rate for patients with COPD was 53.76%, which is consistent with the findings from previous research results. PR is a challenging intervention for patients because it involves the feasibility assessment of PR, the design of clinical and community rehabilitation nursing PR programs, the adherence of patients to rehabilitation behavior, and the support of family members. Therefore, the influencing factors of PR benefits can be considered in the preliminary evaluation, and if there are risk factors, intervention should be performed before rehabilitation which will be conducive to improving the clinical rehabilitation benefit rate.

### Lower baseline levels of 6MWD are associated with an increased probability of benefiting from PR

The results of this study indicate a negative correlation between the baseline 6MWD and benefits from PR. An odd ratio (OR) of 0.977 indicates that the lower the baseline 6MWD, the benefits from PR are more likely. That is, for every 10-meter reduction in the baseline 6MWD, the probability of achieving a PR response increases by 23% (1/0.977^10 = 1.23). This is consistent with the stratified analysis results of Costi et al. (33): Compared with the baseline 6MWD > 350 m group, the baseline 201–300 m group (OR = 1.9) and ≤200 m group (OR = 1.5) had a significantly higher benefit probability, suggesting the presence of a “low start-high gain” effect. Patients with frailty follow-up after 6 months found a significant correlation between exercise capacity and the likelihood of benefiting from PR in patients with COPD (34). PR primarily targets improving the physical activity and respiratory functions in patients with COPD. Those with poor baseline sports ability have more room for improvement and are more likely to benefit from targeted exercise interventions. Due to the ceiling effect, patients with COPD with good exercise capacity may reach a plateau state and be unable to further enhance their exercise capacity through self-directed efforts in the short term. That is why targeting patients with poor physical function for PR training is beneficial. Clinical and community nursing staff play a crucial role in supporting patients undergoing PR, especially for those with lower baseline physical function. Key strategies (35) for nursing staff to enhance patient outcomes and promote a positive attitude toward PR include: educating the patients about the benefits of PR, training them, and encouraging them thus developing a positive mental attitude of patient toward PR. This approach can improve patient outcomes, enhance the success rate of PR programs, and empower individuals to actively manage their chronic respiratory condition and improve their overall quality of life. We verified the robustness of group differences and dynamic changes by adjusting for baseline 6MWD. Although the baseline value had a predictive effect on the outcome (*β* = − 0.12), the effect size of the core interaction term (group × time) was only slightly attenuated (4.1 vs. 4.3), indicating that the rehabilitation advantage in the benefit group was not driven by differences in baseline functioning. This finding further supports the use of baseline 6MWD as a patient stratification indicator rather than a confounder.

### Lower benefit rate of PR in patients with chronic pain

34.79% of patients participating in this study were reported to experience chronic pain issues. A study reported that the prevalence of chronic pain in COPD ranged from 21 to 82% (33), and studies report systemic inflammatory state associated with COPD contributed to the development and persistence of pain (36). Patients with COPD mainly experience pain caused by multiple factors such as pulmonary pain, chest discomfort, musculoskeletal problems, anxiety, depression, and drug side effects (37, 38). The lower rehabilitation benefit rate observed in patients with chronic pain undergoing PR can be due to poor physical adaptability and the influence of long-term disease makes it difficult to adapt to the intensity and frequency of PR training. Other major factors lowering the rehabilitation benefit rate are psychological factors such as anxiety and depression (38). Therefore, when carrying out PR for patients with chronic pain and COPD, it is necessary to comprehensively consider their physical and psychological conditions, formulate personalized rehabilitation programs, and provide professional guidance and support to improve their benefit rates.

### Lower benefit rate of PR in patients with sarcopenia

Sarcopenia is defined as a progressive and systemic skeletal muscle disease, characterized by loss of muscle mass and function over time (28). It is estimated that about 5 to 13% of the “healthy” older adults population may experience sarcopenia (39). In patients with COPD, the prevalence of muscle disease ranges from 8.38 to 52.1% (40). This study found that 39.28% of the patients had sarcopenia, which may be related to the older age of the patients included in this study. Sarcopenia in patients with COPD may be caused by multiple factors such as hypoxia, malnutrition, inflammatory response, reduced muscle activity, and drug side effects (39). Due to the reduction of muscle mass, patients with sarcopenia may have poor exercise tolerance and difficulty in adapting to the intensity and frequency of PR training, which will lead to fatigue and discomfort during training and affect the rehabilitation effect, thus being the main reason for observing low rehabilitation benefits in such patients (41). Further, patients with sarcopenia may lack muscle strength and face difficulty in controlling their position, affecting the correctness of pose during PR training thus limiting the training effects. Therefore, given the sarcopenia patients with COPD, it is important to pay special attention to formulating individualized rehabilitation plans, gradually increasing the intensity of training, and providing professional guidance and support, to improve its benefit rate. This study used an equation method to assess skeletal muscle mass, which, despite rigorous validity validation, may underestimate muscle mass heterogeneity. Integration of standardized tools such as BIA/DXA should be prioritized in future multicenter studies. The clinical predictive value of the 50-meter walk test needs to be further validated in a larger sample.

### Higher benefit rate of PR in patients with better economic conditions and higher family care index

A multi-center cross-sectional survey in China suggests that family support and better economic situations contribute to improved rehabilitation outcomes for patients (42). However, the improvement in rehabilitation outcomes is the subjective feeling of patients in this study, and there is no unified standard.

Patients with better economic and family environments often have enhanced lifestyle support (43). Economic stability ensures access to rehabilitation resources (such as protein supplements and Sports bracelet), Economic security reduces cost anxiety and enhances participation in sports (44). These patients also obtain better psychological support, including psychological counseling and psychotherapy, which can help them overcome psychological obstacles in the process of rehabilitation and improve the rehabilitation outcomes (45). In the PR plan, therefore it is essential to recognize the role of family and encourage family members to actively participate in the patient’s rehabilitation process, to improve the effectiveness of rehabilitation. However, the family of patients with poor economic backgrounds should also try their best to provide support and resources, to ensure effective rehabilitation outcomes.

### Trends in 6MWD and SGRQ between the benefit group and the non-benefit group

The results of this study demonstrated that after 9 weeks of rehabilitation, both the 6MWD and SGRQ scores showed improved trends in both groups. The statement is generally consistent with Bishp’s study (46), however, it fails to address the evolving trend observed in both benefit and non-benefit groups. The exercise capacity of the benefit group improved more than that of the non-benefit group from 6 to 9 weeks of rehabilitation, and the SGRQ scores of the benefit group showed greater improvement than those of the non-benefit group at any time point. The possible reasons are as follows: Through systematic rehabilitation training, patients experienced improvements in lung function, muscle strength, and endurance after 6 weeks, resulting in significant improvement in exercise capacity. (Inferences about lung-function improvement in our study were based on optimization of the response to exercise ventilation rather than on direct spirometry). The improvement in the SGRQ scores of the benefit group at any time point may be attributed to the positive effects of rehabilitation training on the quality of life and psychological state of patients. Thus, rehabilitation training can not only improve the physical condition of patients but also enhance their self-confidence and psychological state. It helps in reducing anxiety and depression, leading to an overall improvement in their quality of life and mental health.

Compared with other studies, the Hafner study (47) focused on the physiological characteristics of patients, suggesting that a more muscular body composition and higher ability to deliver oxygen from the blood to the muscles may be beneficial for PR outcomes. Ragaselvi (6) and Crisafulli (7) reported Osteoporosis was independently associated with poorer recovery outcomes, but the current study did not confirm this finding. BMI had no impact on the patient’s recovery benefits, which is consistent with most research findings (47, 48). It is important to note that this study did not systematically assess sedentary behavior or levels of daily physical activity. Existing evidence suggests that sedentary time is significantly associated with decreased exercise tolerance in patients with COPD, which may affect PR effect by reducing muscle metabolic fitness. “Although we indirectly reflected functional status as measured by baseline 6MWD, future studies are needed to integrate objective monitoring tools such as accelerometers to quantify the dynamic impact of sedentary behavior on PR response. The reliance on self-reported exercise intensity in tele-rehabilitation lacks validation against accelerometer data. This may introduce measurement bias, as COPD patients typically overestimate daily step counts (49). Future studies must integrate wearable sensors (e.g., ActiGraph GT9X) to objectively quantify sedentary behavior and its dynamic impact on PR response.

## Conclusion

The results of this study showed that in patients with clinically stable COPD, the PR benefit rate was low. Baseline 6MWD, chronic pain, and sarcopenia were negatively correlated with PR benefit, while financial situation and better family care index were positively correlated with PR benefit. To improve the stability of patients with COPD’ benefit rate from PR, clinical medical personnel, under the condition of limited resources, can prioritize PR for individuals who are more likely to benefit, to improve the efficiency of clinical PR. For a non-benefit group of patients, it is important to formulate relevant solutions or consider adjusting the recovery cycle to enhance their outcomes. However, this study also has some limitations. First of all, the non-completer group had a higher proportion of severe COPD. This suggests patients with greater disease severity may be more likely to dropout due to exacerbations or functional limitations, necessitating caution when generalizing findings to advanced COPD populations. Relevant biochemical indicators could be considered in subsequent studies to improve the research content. Second, this study was a single-center prospective study, and subsequent studies should increase the sample size and expand the number of centers to improve the reliability and generalization of the study. While some patients exhibited domain-specific improvements, our composite endpoint prioritizes clinically meaningful multidimensional recovery. Future pragmatic studies may explore responder/non-responder phenotypes using machine learning approaches.

## Data Availability

The original contributions presented in the study are included in the article/Supplementary material, further inquiries can be directed to the corresponding author.
