# Supplementary material for: Factors influencing the benefits of pulmonary rehabilitation in older adults with chronic obstructive pulmonary disease: a prospective study
Source: Front Public Health. 2025 Sep 17;13:1644607. doi: 10.3389/fpubh.2025.1644607 (PMC12483923; doi:10.3389/fpubh.2025.1644607)
Supplement: Supplementary file 2 [file Table_1.docx]

Supplementary File 1

The exercise therapy protocol included:

​​Aerobic training​: Intensity was individually regulated using the Borg CR-10 scale (target range: 4-6, corresponding to 'somewhat strong' to 'strong' perceived exertion), with concurrent target heart rate monitoring to ensure safety (target zone: [220 − age] × 60%–80%).

​​Progressive resistance training​: Initial intensity was set at 50% of one-repetition maximum (1RM), increased by 10% every 2 weeks based on tolerance.

​​Balance training​: Real-time feedback was provided using computerized dynamic posturography to maintain center-of-pressure sway velocity <2.5°/s."

FITT Parameters​​

Structured training variables using the FITT framework (Frequency, Intensity, Time, Type):

| Component | Aerobic Training | Resistance Training | Balance Training |
| --- | --- | --- | --- |
| Frequency | 3 sessions/week | 2 sessions/week | Daily |
| Intensity​ | Borg CR-10: 4–6 | 60%–80% 1RM | Eyes-closed single-leg stance ≥30s |
| Time | 30–40 min/session | 3 sets × 10 reps | 10 min/session |
| Type | Cycling/treadmill | Elastic bands/machines | Tai Chi steps/balance mats |

Note:1RM measurement process:Calculated by the maximum weight (5RM) of 5 repetitions: 1RM = 5RM × 1.2; The initial load was set at 50% of 1RM, adjusted according to Borg score

**Dynamic Adjustment Protocol​​**

Progression criteria(meet any):

- Borg score <4 for three consecutive sessions
- 6MWD improvement ≥15 meters/week
- SpO₂ decline <3% during training

Regression criteria (immediate action):

- SpO₂ <88% for >1 minute
- Borg score >7 with dyspnea exacerbation
- BP >180/105 mmHg

Supplementary Table S1. Missing Data Patterns​

| Assessment Timepoint | 6MWD Missing (n) | SGRQ Missing (n) | Primary Reason |
| --- | --- | --- | --- |
| Baseline | 2/254 (0.8%) | 1/254 (0.4%) | Equipment malfunction |
| 3-week | 8/196 (4.1%) | 6/196 (3.1%) | Acute exacerbation (n=5) |
| 6-week | 14/196 (7.1%) | 11/196 (5.6%) | Loss to follow-up (n=9) |
| 9-week | 9/196 (4.6%) | 8/196 (4.1%) | Transportation barriers (n=6) |
